# Oligoarginine Peptide Conjugated to BSA Improves Cell Penetration of Gold Nanorods and Nanoprisms for Biomedical Applications

**DOI:** 10.3390/pharmaceutics13081204

**Published:** 2021-08-05

**Authors:** Karen Bolaños, Macarena Sánchez-Navarro, Andreas Tapia-Arellano, Ernest Giralt, Marcelo J. Kogan, Eyleen Araya

**Affiliations:** 1 Departamento de Química Farmacológica y Toxicológica, Facultad de Ciencias Químicas y Farmacéuticas, Universidad de Chile, Santiago 8380494, Chile; atare_28@ciq.uchile.cl; 2 Laboratorio de Comunicaciones Celulares, Centro de Estudios en Ejercicio, Metabolismo y Cancer (CEMC), Santiago 8380453, Chile; 3 Advanced Center of Chronic Diseases (ACCDis), Santiago 8380494, Chile; 4 Institute for Research in Biomedicine (IRB Barcelona), The Barcelona Institute of Science and Technology, Baldiri Reixac 10, 08028 Barcelona, Spain; macarena.sanchez@ipb.csic.es (M.S.-N.); ernest.giralt@irbbarcelona.org (E.G.); 5 Department of Inorganic and Organic Chemistry, University of Barcelona, Martí i Franquè s 1–11, 08028 Barcelona, Spain; 6 Departamento de Ciencias Quimicas, Facultad de Ciencias Exactas, Universidad Andres Bello, Santiago 8370146, Chile

**Keywords:** cell internalization, albumin, BSA, CPP, gold nanorods, gold nanoprisms, arginine-rich peptide

## Abstract

Gold nanoparticles (AuNPs) have been shown to be outstanding tools for drug delivery and biomedical applications, mainly owing to their colloidal stability, surface chemistry, and photothermal properties. The biocompatibility and stability of nanoparticles can be improved by capping the nanoparticles with endogenous proteins, such as albumin. Notably, protein coating of nanoparticles can interfere with and decrease their cell penetration. Therefore, in the present study, we functionalized albumin with the r_8_ peptide (All-D, octaarginine) and used it for coating NIR-plasmonic anisotropic gold nanoparticles. Gold nanoprisms (AuNPrs) and gold nanorods (AuNRs) were coated with bovine serum albumin (BSA) previously functionalized using a cell penetrating peptide (CPP) with the r_8_ sequence (BSA-r_8_). The effect of the coated and r_8_-functionalized AuNPs on HeLa cell viability was assessed by the MTS assay, showing a low effect on cell viability after BSA coating. Moreover, the internalization of the nanostructures into HeLa cells was assessed by confocal microscopy and transmission electron microscopy (TEM). As a result, both nanoconstructs showed an improved internalization level after being capped with BSA-r_8_, in contrast to the BSA-functionalized control, suggesting the predominant role of CPP functionalization in cell internalization. Thus, our results validate both novel nanoconstructs as potential candidates to be coated by endogenous proteins and functionalized with a CPP to optimize cell internalization. In a further approach, coating AuNPs with CPP-functionalized BSA can broaden the possibilities for biomedical applications by combining their optical properties, biocompatibility, and cell-penetration abilities.

## 1. Introduction

Research on nanomaterials has expanded in recent years for their use in drug delivery, imaging, therapy, diagnosis, and combined therapy, among other fields [1,2,3,4,5]. Organic, inorganic, biological-type, or hybrids between different structures have been proposed for diverse medical/biomedical applications [6,7,8,9,10,11,12,13,14]. Among the prospective materials for future applications, gold nanoparticles stand out, owing to their wide-ranging potential. 

AuNPs have characteristic optical properties that are derived from their localized surface plasmon resonance (LSPR), allowing them to interact with light in a different way than bulk materials do [10,15,16]. The plasmon excitation has two main decay mechanisms: radiative and non-radiative, resulting in light scattering and absorption, respectively [17]. Light scattering of AuNPs has been extensively used to design diagnostic tools, contrast agents, and Raman enhancement probes [18,19,20]. Conversely, absorption of light involves relaxation via electron-electron collisions or electron-lattice-phonon couplings, yielding light-to-heat conversion, [21] referred to as the photothermal effect, which can be used for drug release and photodynamic and photothermal therapy [22].

Both LSPR and the optical properties of AuNPs are highly shape- and size-dependent. In this regard, AuNRs and AuNPrs are two interesting AuNP geometries because their light absorption can be synthetically modulated. Therefore, their maximum absorption can be tuned to the biological window (the region that exhibits minimum light absorption of the biological tissues) [23,24,25,26], with a strong optical extinction and for photothermal applications [17,27].

Given the wide range of applications for AuNPs as biomedical platforms, it is essential to consider the biocompatibility of this material. AuNPrs (synthesized by sodium thiosulfate reduction) have been shown to be non-cytotoxic [28,29]. In contrast, AuNRs are commonly synthesized using the CTAB surfactant, which has shown some cytotoxic effects [30,31,32].

In order to avoid possible cytotoxic effects from our nanocarriers, we incorporated a protein coating on both AuNPs to increase their biocompatibility and stability [6,29,33,34,35,36]. BSA shares 76% sequence identity with human serum albumin (HSA) [37] and is well tolerated by humans [38]. This protein has been extensively used for the development of nanomaterials for biomedical applications in drug delivery, therapy (photothermal or combined), diagnosis, and theranostics [39,40,41,42,43,44,45].

Even though the presence of proteins as a coating on the nanoparticle’s surface improves their biocompatibility and stability properties, it may also limit their internalization capacity due to alterations of the protein corona composition and, consequently, the interaction with receptors and membranes [46,47,48]. In this regard, the possible biomedical applications of AuNPs can be expanded as their cell internalization ability is increased. Accordingly, arginine-rich CPPs, (minimum amount of six Arg) are well known for their ability to cross biological barriers [49,50]. Although other studies have reported the conjugation of nanoparticles with internalization peptides in a direct way [51,52,53,54], it is also well known that the presence of BSA improves the circulation time of nanoparticles and controls the composition of the protein corona in physiological media, hence the importance of the inclusion of albumin in nanoparticles [37,55,56]. Therefore, we functionalized the BSA protein with r_8_ to facilitate cell penetration of the AuNPs. Previous studies have shown that the use of arginine-rich CPPs increases cell penetration of the nanocarriers, opening the possibility for the improved cell internalization of AuNPs [51,57,58,59]. In particular, r_8_ has been covalently linked to several molecules and nanosystems, such as insulin [60] liposomes, [61] quantum dots, [62] and gold nanorods, [49,58] improving their uptake and cell penetration in target sites. Nevertheless, coating AuNRs and AuNPrs (with absorption in the first biological window) with BSA, functionalized with r_8_ for cell internalization, has not been reported yet. In this study, we proposed that BSA-r_8_ enhances the cell internalization of AuNPs, taking as examples two promissory anisotropic AuNPs with different surface and charge: AuNRs and AuNPrs, with absorption on the first biological window (650–950 nm) [63] for possible applications in biomedical nanoplatforms.

## 2. Materials and Methods

### 2.1. Materials

HAuCl_4_ (Gold (III) chloride hydrate), Na_2_S_2_O_3_ (sodium thiosulfate), hexadecyltrimethylammonium bromide (CTAB), NaBH_4_, and AgNO_3_ were acquired from Sigma-Aldrich (St. Louis, MO, USA). Polyethylene Glycol 5 kDa (HS-PEG-COOH, 5 kDa) was from JenKem Technology (Beijing, China). Milli-Q water was obtained from the purification of distilled water with the Simplicity SIMS 00001 equipment (Millipore, Molsheim, France). Cell culture plates and flasks were from Corning Costar (Corning, NY, USA). Penicillin/streptomycin and chemicals for cell culture were from Gibco (Gibco-BRL, Paisley, UK), fetal bovine serum (FBS, Biological Industries, Cromwell, CT, USA). MTS/PMS [3-(4,5-dimethylthiazol-2-yl)-5-(3-carboxymethoxyphenyl)-2-(4-sulfophenyl)-2H-tetrazolium, inner salt, MTS/phenazine methosulfate, PMS], and CellTiter 96 kit were from Promega (Promega, Madison, WI, USA). Atto-565 NHS ester (A565) was from Sigma (Sigma-Aldric Chemie, Buch, Switzerland). Other reagents were from Sigma-Aldrich. 

### 2.2. Experimental

#### 2.2.1. AuNRs Synthesis

AuNRs were synthesized using a previously reported seed-mediated procedure [64]. Briefly, 5 mL of a 0.3 mM HAuCl_4_ solution in CTAB 0.1 M was reduced by ice-cold NaBH_4_ 10 mM (300 µL), resulting in a brownish-yellow seed solution. Then, 10 mL of a 0.5 mM HAuCl_4_ solution (in CTAB 0.1 M) was reduced by ascorbic acid in the presence of AgNO_3_, until it reached a colorless growth solution. Finally, 120 µL of the seed solution was added to the growth solution and allowed to rest for 30 min in a thermostatic bath at 27 °C. The obtained AuNRs were centrifuged at 7030 g for 30 min, and the pellet was resuspended in Milli-Q water. 

#### 2.2.2. AuNPrs Synthesis

AuNPrs were obtained by Na_2_S_2_O_3_ reduction of HAuCl_4_, as previously reported [28,36]. A 2 mM HAuCl_4_ solution was first reduced by 0.6 mM Na_2_S_2_O_3_ and allowed to rest for 9 min. Then, a second addition of 0.6 mM Na_2_S_2_O_3_ was performed, and the solution was left undisturbed for 30 min. The purple solution containing the AuNPs was centrifuged and resuspended in Milli-Q water. PEG functionalization was achieved at pH = 12 by adding 15 µL of a 2.7 mM HS-PEG5000-COOH solution and allowing conjugation under a magnetic stirrer for 3 h. Finally, AuNPrs were separated from smaller undesired AuNPs using a successive differential centrifugation procedure, as reported [36].

#### 2.2.3. Characterization of the Nanoparticles 

AuNRs and AuNPrs were characterized before and after BSA-r_8_ coating by UV-Vis-NIR absorption spectra, using a Lambda 25 spectrophotometer (Perkin Elmer, Waltham, MA, USA). Dynamic light scattering (DLS) and Z potential measurements were acquired in PBS pH = 7, at 25 °C, using a Zetasizer 3000 (Malvern Instruments, Malvern, UK), as triplicates in aqueous solution at 25 °C. 

TEM images of AuNRs were acquired with a JEOL JEM-1010 microscope (JEOL USA, Peabody, MA, USA), using Formvar carbon-coated copper microgrids (200 mesh; Ted Pella, Redding, CA, USA). For AuNPrs, TEM images were obtained using a Philips CM 120 transmission electron microscope with an accelerating voltage of 120 kV and a 300 mesh Formvar/Carbon-Coated Copper grid. For both AuNPs, liquid suspensions were deposited on the microgrid and allowed to stand overnight before TEM image acquisition. 

### 2.3. Peptide Synthesis

We synthesized the all-D peptide derived from D-amino acids for this study due to their enhanced enzymatic stability [65]. Arginine-rich peptide (r_8_) was synthesized by solid phase peptide synthesis (SPPS), using an H-Rink Amide Protide resin (loading: 0.56 mmol/g) in a Liberty Blue™ Automated Microwave Peptide Synthesizer. Linear D-OctoArginine was synthesized on a 0.5 mmol scale using a 5 excess of Fmoc-amino acid (0.2 M), relative to the resin. The Bromo acetic acid was coupled using 2 cycles of 30 min of 4 equivalents of OxymaPure, followed by 4 equivalents of N,N′-Diisopropylcarbodiimide, and then 4 equivalents of bromo acetic acid. Fmoc deprotection was carried out using 10% (*w*/*v*) piperazine and 0.1 M OxymaPure in a 9:1 mixture of NMP and EtOH. The resin was cleaved using TFA/H_2_O/TIS (95%/2.5%/2.5%) for 6 h; the TFA was evaporated, dissolved in a 50/50 H_2_O/ACN solution, and lyophilized. The peptide was purified by semi-preparative HPLC on a Waters 2700 sample manager, equipped with a Waters 2487 dual-wavelength absorbance detector, a Waters 600 controller, a Waters fraction collector and Masslynx software by using a Sunfire C18 column (150 × 10 mm × 3.5 μm, 100 Å, Waters), flow rate 6.6 mL/min; solvent A = 0.1% TFA in water and solvent B = 0.1% TFA in acetonitrile. Purity and identity were assessed by UPCL (Waters Acquity equipped with Acquity photodiode array detector, flux rate 0.610 mL/min, Acquity UPLC BEH C18 Column, 130 Å, 1.7 µm, 2.1 mm × 100 mm; solvents A = 0.045% TFA in water, and B = 0.036% TFA in acetonitrile) and UPLC-MS (Waters Acquity UPLC System equipped with ESI-SQ Detector2, flux rate 0.610 mL/min, Acquity UPLC BEH C18 Column, 130 Å, 1.7 µm, 2.1 mm × 100 mm; solvents A = 0.1% formic acid in water and B = 0.1% formic acid in acetonitrile).

The crude compound was purified by RP-HPLC at a semi-preparative scale and characterized by UPLC and UPLC-MS spectrometry (Supplementary Section 1, Figures S1 and S2) to confirm the identity of the synthesized compound, obtaining high purity (>95%). Amino acid content was analyzed by amino acid analysis; results are presented in Supplementary Section 2, Table S1.

### 2.4. BSA Functionalization

**Scheme 1:**
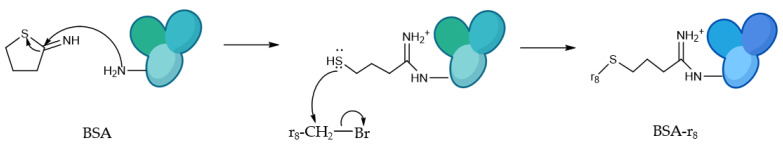
Synthetic approach for BSA functionalization by r_8_ peptide. First, 2-iminothiolane reacts with BSA amine groups in the surface of the protein, giving rise to available SH free groups, followed by r_8_-Br reaction with the free SH groups.

BSA was functionalized with r_8_, as represented in Scheme 1, to improve the internalization properties of the nanoconstructs, and Atto-565-NHS-ester was used as a fluorescent probe for detection by fluorescence and confocal microscopy. 

#### BSA Labeling with r_8_ and Atto 565

- First step: 2-Iminothiolane functionalization

A 100 mg/mL 2-Iminothiolane solution (15 µL) was added to a 10 mg/mL BSA solution (50 mg of BSA in 5 mL of PBS), in 4 intervals every 10 min (molar ratio 10:1 2-iminothiolane: BSA), and the reaction was performed for 1 h at 4 °C. The non-reacted 2-iminothiolane was removed using a P10 G25 desalting column from Sigma-Aldrich.

- Second step: r_8_ peptide functionalization

To the previously prepared protein, 52 µL of a 100 mg/mL Br-CH_2_-r_8_ solution was added in 4 intervals every 10 min, and the reaction was left overnight at 4 °C (ratio 5:1 r_8_-Br:BSA). The product was purified with a P10 G25 desalting column and analyzed by UV-Vis-NIR spectroscopy and amino acid analysis to determine the amount of r_8_ per BSA molecule.

- Third step: amino acid analysis of BSA-r_8_

Amino acid analysis was performed following an acid hydrolysis procedure, adding 12 N HCl and a known concentration standard (ϒ-aminobutyric acid, 0.1 mM), and allowed to react at 110 °C for 72 h. Amino acid quantification was carried out in an HPLC-PDA AccQ-Tag (C18; 4 μm; 3.9 × 15 mm). The amino acid content on the functionalized BSA resulted in a 2.4 r_8_/BSA ratio, as shown in Supplementary Section 2, Table S1.

- BSA-r_8_ fluorescent labeling

**Scheme 2:**
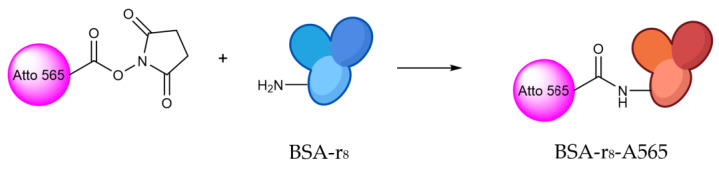
Synthetic approach for BSA-r_8_ functionalization by Atto565-NHS ester in the NH_2_ free groups of BSA.

Fluorescent labeling of BSA was conducted as represented in Scheme 2. To 10 mg of BSA-r_8_ of the previous step, 10.7 µL of a 10 mg/mL solution of Atto 565 NHS ester was added and allowed to react for 1 h. The final product (BSA-r_8_-A565) was purified with a P10 G25 desalting column and characterized by UV-Vis spectroscopy. 

According to UV-Vis spectroscopy measurements, the degree of labeling (DOL) was calculated as follows:

$$DOL=\frac{Absorbance\;565\times \varepsilon_{Atto\;565}}{Absorbance\;280\;\left(Absorbance\;565\times CF_{Atto\;565}\;\right)\times \varepsilon_{BSA}}$$

where:

*Ɛ_Atto_*_565_ = 1.2 × 10^5^ M^−1^ cm^−1^, *Ɛ_BSA_* = 4.6 × 10^5^ M^−1^ cm^−1^, and correction factor Atto 565 (*CF_Atto_*_565_) = 0.16

As a result, the DOL of BSA-r_8_ A565 was 0.66 mol Atto/mol BSA. 

### 2.5. Circular Dichroism

The BSA and BSA-r_8_ 1.5 × 10^−5^ M samples were prepared in PBS 0.1× in the same conditions. The spectra were acquired at 20 °C, recorded from 200 to 250 nm in triplicate and condensed into a single spectrum to reduce noise at a 1 nm/s rate in a JASCO J-815 instrument (JASCO, Easton, MA, USA), using a 1 mm pathlength quartz-cuvette. The secondary structure content was calculated using the CDPro CONTIN 2DP (AUG 1982) (2DP-SW PACK) version from JASCO.

The molar ellipticity at wavelength λ ([θ_mrw_]) was calculated as follows:

$$\left[\theta_{\mathrm{mrw}}\right]=\frac{MRW\times \theta_{\lambda }}{10\times d\times c}$$

where *θ_λ_* is the observed ellipticity (degrees) at wavelength λ, *d* is the pathlength (cm), and *c* is the protein concentration (g/mL) [66]. 

### 2.6. Capping of AuNPs with BSA or BSA-r_8_

BSA-r_8_ or BSA capping of the AuNPs was achieved by incubation, as previously reported [36]. Briefly, to 1 mL of AuNRs or AuNPrs at a 1 nM concentration, a solution of BSA or BSA-r_8_ was added (final concentration of BSA = 1 mg/mL in 0.1× PBS), incubating for 2 h at 4 °C in low-binding 1.5 mL centrifuge tubes (Eppendorf^®^, Hamburg, Germany), and the samples were centrifuged at 10,000 rpm for 10 min. The pellet containing the BSA or BSA-r_8_ coated AuNPs was resuspended in 0.1× PBS. The protein content was quantified on the supernatants using a Micro BCA™ Protein Assay Kit from Thermo Scientific™ (Pierce, Rockford, IL, USA), according to the manufacturer’s specifications, in triplicate.

### 2.7. Cell Culture

HeLa cells were cultured in complete DMEM, containing 1% penicillin/streptomycin, 10% FBS and 5% glutamine. The culture was maintained at 37 °C and 5% CO_2_. 

#### 2.7.1. Cell Viability MTS Assays

HeLa cells (1 × 10^4^ cells/well) were seeded in TC pretreated 96-well plates and allowed to attach at 37 °C for 24 h. The treatments were added and allowed to incubate for 24 h. The medium was then replaced by a phenol red-free medium containing the MTS/PMS containing reagent, following the manufacturer’s recommendations. The absorbance of the culture medium after incubation for 1 h was recorded at 490 nm using a microplate reader. Cell viability was calculated with respect to a non-treatment control (live control). Each treatment and control were made in quintuplicate, minimum *n* = 3. Statistical analysis was performed using GraphPad Prism V5.01. 

#### 2.7.2. Confocal Microscopy

HeLa cells (3 × 10^5^ cells/plate) were seeded on collagen pre-coated MatTek glass-bottom culture dishes (MatTek Corporation, Ashland, MA, USA) and attached for 48 h at standard culture conditions. The medium was then replaced with fresh medium, and the treatments were added considering 1 nM of each AuNP and incubated (1 or 24 h of treatment). The cells were subsequently washed 3 times with PBS, and phenol red-free medium containing the LIVE/DEAD^®^ Cell Imaging Kit from ThermoFisher was added (Molecular Probes Inc., Eugene, OR, USA). Fluorescence was detected on a Zeiss LSM 880 laser scanning microscope (Zeiss, Berlin, Germany) with Airyscan, equipped with a CO_2_ and temperature-controlled environmental chamber. Hoechst was excited with an Ar laser at 405 nm, and emission was recorded at 458 nm; Atto-565 was excited by a laser at 405 nm, and emission was recorded at 458 nm. Images were processed using Image J 1.52 p software. 

#### 2.7.3. Internalization Evaluated by Transmission Electron Microscopy

In a Petri dish (90 × 15 mm), 1 × 10^6^ HeLa cells were seeded and incubated at 37 °C until a minimum confluence of 80%. The cells were then washed, the medium was replaced with a treatment containing-medium (1 nM of BSA or BSA-r_8_ coated AuNPs), and the cells were incubated for 24 h. Afterwards, the cells were washed and fixed with glutaraldehyde 2.5% in PBS 0.1×. The cells were scraped and centrifuged, and the obtained pellet was fixed with OsO_4_ 1% in PBS for 90 min and embedded in Epon resin. The resin was sliced into slices of 80 nm thickness, and the slices were placed in a Cu grid and stained with Reynold’s reagent and uranyLess staining kit, before visualization. TEM images were acquired in a JEOL JEM-1010 microscope (JEOL Ltd., Tokyo, Japan).

## 3. Results and Discussion

### 3.1. Preparation of AuNRs-BSA-r_8_ and AuNPr-BSA-r_8_

**Figure 1:**
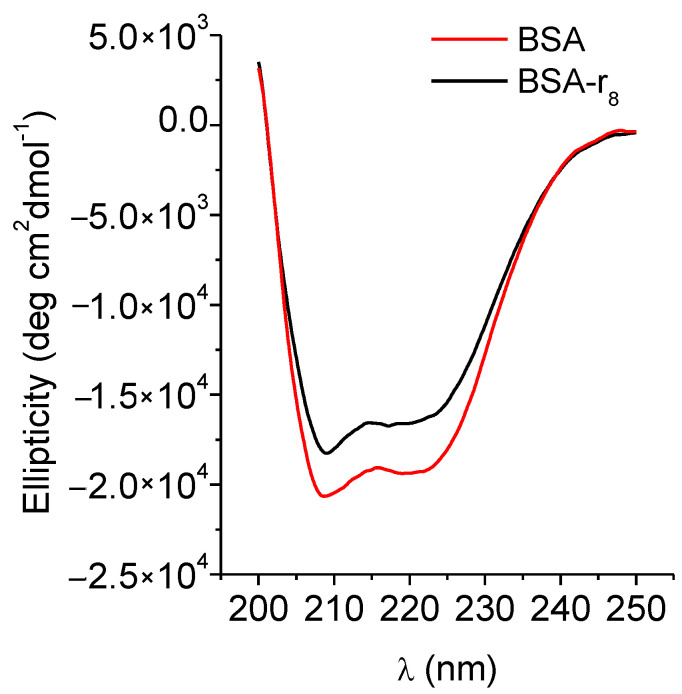
Circular dichroism spectrum of BSA before and after r_8_ functionalization. DOL: 2.4 r_8_/BSA. BSA and BSA-r_8_ 1.5 × 10^−5^ M samples were prepared in PBS.

The r_8_ peptide was synthesized and characterized by UPLC-MS, showing the characteristic [M+2H]^2+^ = 695.02 m/z (Figure S1). Then, BSA was functionalized with r_8_ (2.4 r_8_/BSA) as described in the experimental section. The degree of BSA functionalization was determined by amino acid analysis, resulting in 2.4 r_8_/BSA. To determine if the functionalization of the protein led to a change in BSA secondary structure, circular dichroism spectra were obtained. Figure 1 shows the CD spectrum of BSA and BSA-r_8_, indicating that they shared a similar profile, with a non-significant change from 39% and 36% alpha helix content before and after r_8_ functionalization, respectively.

BSA-r_8_ was functionalized with a fluorescent probe, namely A565, a known and commonly used red fluorescent probe, obtaining a 0.66 A565/BSA-r_8_ degree of labeling, which resulted in BSA-A565-r_8_. In this study, we tested whether AuNPrs were a suitable alternative to more-traditional AuNRs; in this sense, AuNPrs were synthesized by simply reducing Au^3+^ with Na_2_S_2_0_3_, thus avoiding the involvement of cytotoxic agents [28]. AuNRs and AuNPrs were synthesized with a characteristic morphology, as TEM images show in Supplementary Section 3 (Figures S3 and S4), with a predominant aspect ratio of 3.5 for AuNRs and a 60 nm edge-length for AuNPrs.

**Figure 2:**
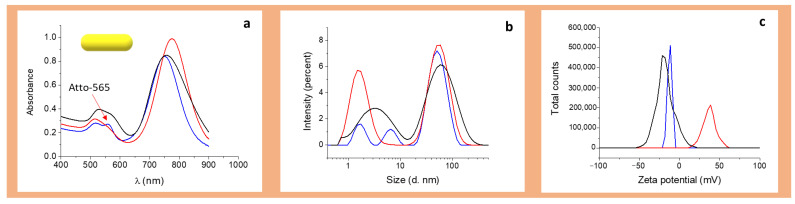
Characterization of BSA-A565-r_8_ capped AuNRs: red line, AuNRs, black line AuNR-BSA, blue line AuNR-BSA-A565-r_8_. (**a**) UV-Vis NIR spectra. The red arrow shows the presence of Atto-565. (**b**) Size distribution. (**c**) Zeta potential distribution. Acquired in PBS, pH = 7.

**Figure 3:**
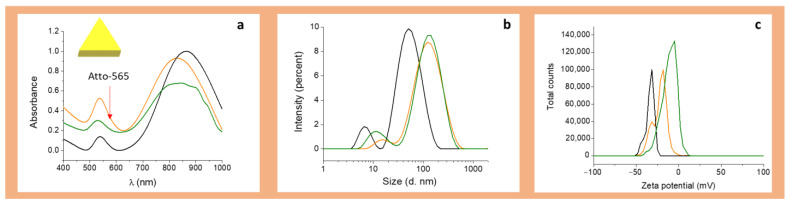
Characterization of BSA-A565-r_8_ capped AuNPrs; black line AuNPrs, green line AuNPr-BSA, and orange line AuNPr-BSA-A565-r_8_. (**a**) UV-Vis-NIR spectra. The red arrow shows the presence of Atto-565. (**b**) Size distribution. (**c**) Zeta potential distribution. Acquired in PBS, pH = 7.

It has been demonstrated by several spectroscopic techniques that BSA is spontaneously adsorbed in the surface of AuNPs via S-Au bonds between the free SH groups of BSA and the gold atoms in the AuNP surface [42,67,68,69,70,71]. Therefore, both AuNRs and AuNPrs were coated with BSA and the functionalized BSA-A565-r_8_ by incubation, as summarized in Table 1 and Figure 2 and Figure 3. Figure 2 and Figure 3 show the UV-Vis-NIR spectra, size, and Z potential of the BSA and BSA-A565-r_8_ functionalized AuNPs in PBS (pH = 7). For AuNRs, a shoulder around 558 nm appeared after BSA-A565-r_8_ protein coating (Figure 2a), as well as an increment in the intensity of the first plasmon in AuNPrs (Figure 3a), confirming the presence of A565 and therefore, the functionalization of BSA on the AuNPs surface. In addition, the second plasmon of both AuNPs was shifted after the BSA and BSA-r8-A565 coating, attributed to the modification in the dielectric constant of the AuNPs [72] (Table 1 and Figure 2a and Figure 3a), due to the presence of the protein on the AuNPs surface, as reported in previous studies [36,73,74,75].

For both nanoparticles, the protein coating on the surface increased the hydrodynamic diameter in similar proportions using either the BSA or BSA-A565-r_8_, as expected for the presence of the protein (Table 1 and Figure 2b and Figure 3b). For AuNRs, hydrodynamic diameter increased from 2 ± 1, 59 ± 3 nm to 3 ± 1, 68 ± 3 nm and 6 ± 1, 79 ± 3 nm after the BSA and BSA-A565-r_8_ coating, respectively. Meanwhile, for AuNPrs, the hydrodynamic diameter shifted from 4 ± 1, 68 ± 4 nm to 10 ± 2, 142 ± 5 nm and 12 ± 2, 142 ± 5 nm due to the BSA and BSA-A565-r_8_ coating, respectively. 

Regarding the Z potential, a similar trend was observed, and both nanoparticles showed similar Z potentials after the BSA or BSA-A565-r_8_ coating. AuNRs showed an initial Z potential of +45 ± 3 mV, due to the presence of the cationic surfactant CTAB [76,77]; in contrast, AuNPrs had an initial Z potential of −31 ± 3 mV, due to the stabilizing agent HS-PEG-COOH on the surface [36]. Then, the Z potential exhibited a shift to negative values; −21 ± 1 and −17 ± 1 for AuNR-BSA and AuNR-BSA-A565-r_8_ and −14 ± 1 and −18 ± 1 mV for AuNPr-BSA and AuNPr-BSA-A565-r_8_, respectively (Table 1 and Figure 2c and Figure 3c), due to the negative charge of BSA (pI_BSA_: 4.5–5.0) [78] on the surface of both AuNPs. Notably, AuNPs exhibited similar negative Z potentials, with adequate values to interact with cell membranes for internalization [60,61,62]. Stability of AuNR-BSA-r_8_ and AuNPr-BSA-r_8_ was assessed 30 days after storage at 4 °C; DLS and Z potential did not show significant differences over that time (Supplementary Section 4, Figure S5).

### 3.2. Cell Viability Assays

#### 3.2.1. Effect of AuNRs and AuNRs-BSA-A565-r_8_ on Cell Viability

**Figure 4:**
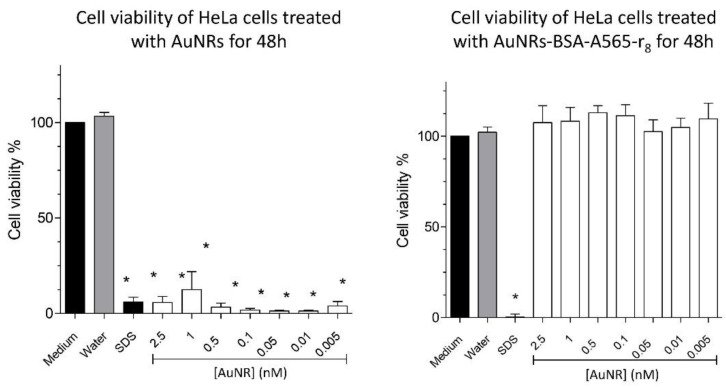
Effect of AuNRs and AuNR-BSA-r_8_ incubated for 24 h on cell viability of HeLa cells at 48 h. The average values for *n* = 4 experiments are shown with error bars representing the SEM. * Significant difference according to Tukey’s test *p* < 0.05 compared to medium control.

One of the main limitations of using AuNRs in biomedical applications is the cytotoxicity associated with the CTAB on the AuNP surface as the stabilizing agent in the synthesis procedure [30]. In order to overcome this issue, AuNRs can be coated with different materials, such as BSA [39,79,80,81,82] to increase their biocompatibility. In this regard, Figure 4 shows that the freshly synthesized AuNRs drastically decreased the viability of Hela cells, whereas AuNRs-A565-BSA-r_8_ did not cause a significant effect between the 0.005–2.5 nM range at 48 h. Additionally, flow cytometry showed no effect of 1 nM AuNRs-A565-BSA-r_8_ after 24 h of administration (Supplementary Section 5, Figure S6). Similar results were found for BSA-coated AuNPs in previous studies; the BSA coating on AuNRs improved their biocompatibility properties [83,84,85], highlighting the potential of coated AuNRs for bioapplications.

#### 3.2.2. Effect of AuNPrs and AuNPrs-BSA-r_8_ on Cell Viability

**Figure 5:**
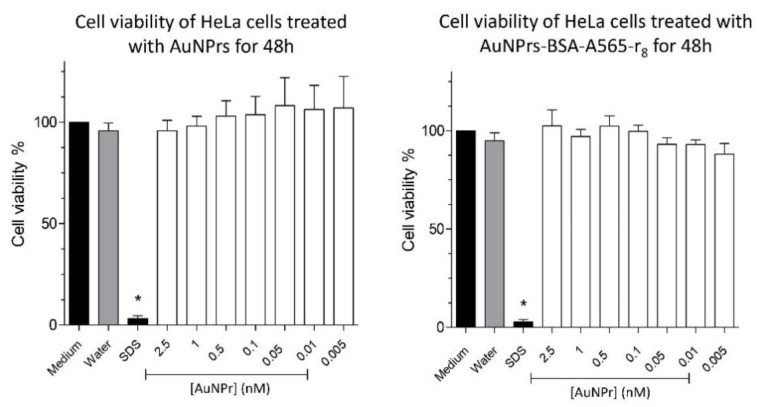
Effect of AuNPrs and AuNPr-BSA-r_8_ incubated for 24 h on cell viability of HeLa cells at 48 h. The average values for *n* = 4 experiments are shown with error bars representing the SEM. * Significant difference according to Tukey’s test *p* < 0.05 compared to medium control.

AuNPrs were functionalized with PEG-COOH to increase their colloidal stability [86] and subsequently coated with BSA-A565-r_8_ (AuNPr-BSA-A565-r_8_). Figure 5 shows cell viability of HeLa cells treated with AuNPrs and AuNPr-BSA-A565-r_8_ at the 0.005–2.5 nM range. As expected, neither AuNPrs nor AuNPrs-BSA-r_8_ showed any effect on HeLa cell viability after 48 h of treatment by MTS assay. Flow cytometry also showed non-effect of AuNPrs-BSA-r_8_ 1 nM in the HeLa cells after 24 h of incubation (Supplementary Section 5, Figure S6). This null effect on cell viability of AuNPrs was previously demonstrated in studies such as those of Alfranca et al. and Bao et al., using Vero cells and HT-29 cells at 72 h, respectively [29,87]

### 3.3. Cell Internalization

#### 3.3.1. Confocal Microscopy

**Figure 6:**
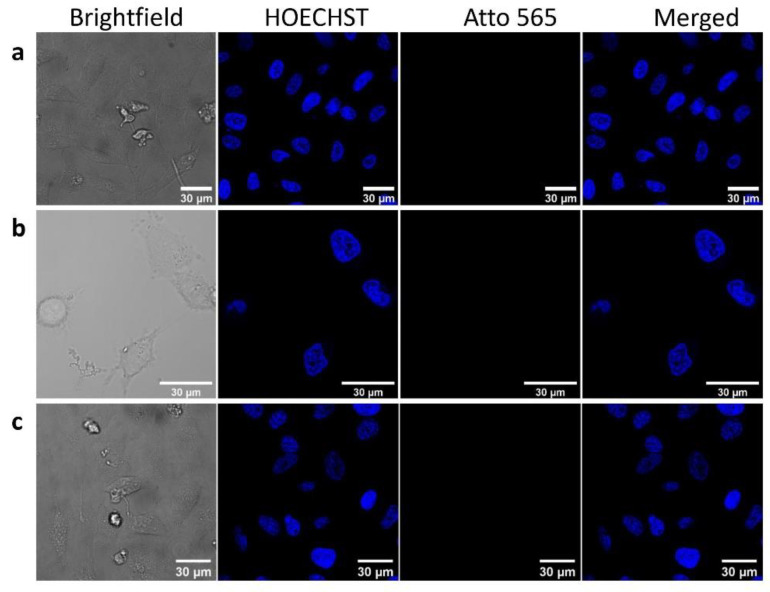
Confocal microscopy images of HeLa cells after treatment by: (**a**) BSA-A565, (**b**) AuNR-BSA-A565, (**c**) AuNPr-BSA-A565 incubated for 24 h. Scale 30 µm. [BSA] = 5 µM, [AuNR-BSA, AuNPr-BSA] = 1 nM. Channels: bright blue (HOECHST), red (Atto-565), blue and red (merged).

In a first step, we tested the internalization ability of the AuNPs capped with non-CPP functionalized BSA, as shown in Figure 6. The comparison between the BSA, AuNR-BSA, and AuNPr-BSA (labeled with A565) signals showed that BSA on the AuNP surface was not able to promote cell uptake under the studied conditions ([BSA] = 5 µM, [AuNR-BSA and AuNPr-BSA] = 1 nM, 24 h of incubation), as demonstrated by the absence of signal in the red channel.

**Figure 7:**
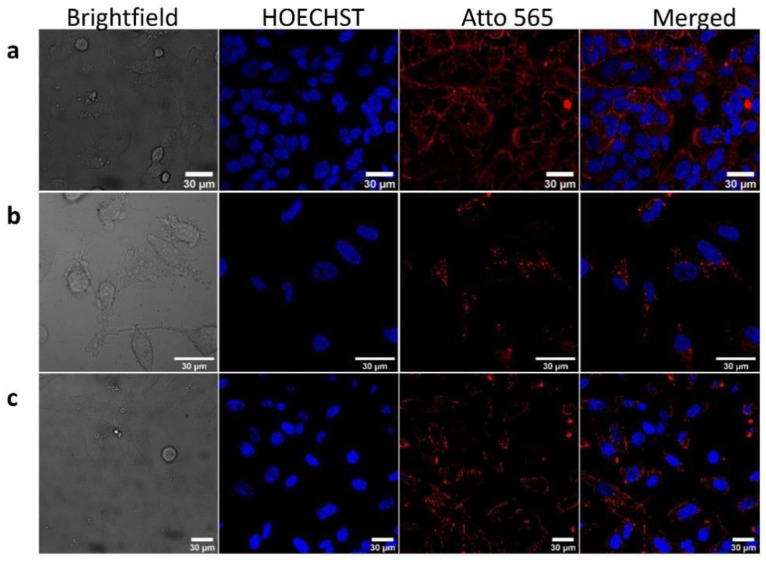
Confocal microscopy images of HeLa cells after treatment with: (**a**) BSA-r_8_-A565, (**b**) AuNR-BSA-r_8_-A565, (**c**) AuNPr-BSA-r_8_-A565 incubated for 24 h. Scale 30 µm. [BSA-r_8_] = 5 µM, [AuNR-BSA-A565-r_8_ and AuNPr-A565-BSA-r_8_] = 1 nM. Channels: bright blue (HOECHST), red (Atto-565), blue and red (merged).

Internalization of BSA-r_8_ and the AuNPs capped with BSA-r_8_ (labeled with A565) was subsequently assessed by confocal microscopy. As Figure 7 shows, functionalization with r_8_ increased the uptake of the protein, as well as that of the protein-coated AuNRs and AuNPrs in the red channel, using the same established conditions as in Figure 7 ([BSA] = 5 µM, [AuNR-BSA-A565-r_8_] and [AuNPr-A565-r_8_] = 1 nM), suggesting the prevalent role of the r_8_ for cell internalization in both nanoconstructs, regardless of their surface charge.

Although discussing the exact mechanism of internalization of the proposed nanoconstructs was not the objective of our study, previous reports have indicated that nanoconstructs conjugated with CPPs are internalized in vesicles inside cells via endocytic uptake [29,51,59,88], while arginine-rich CPPs can electrostatically bind with the cell membranes, promoting translocation into the cells [29,51,59,88].

To confirm that the internalization observed through confocal microscopy corresponded to the functionalized AuNPs, and to elucidate the intracellular location of the nanoconstructs in Hela cells, we performed a TEM study, as indicated in the next section. 

#### 3.3.2. TEM for Cell Internalization

**Figure 8:**
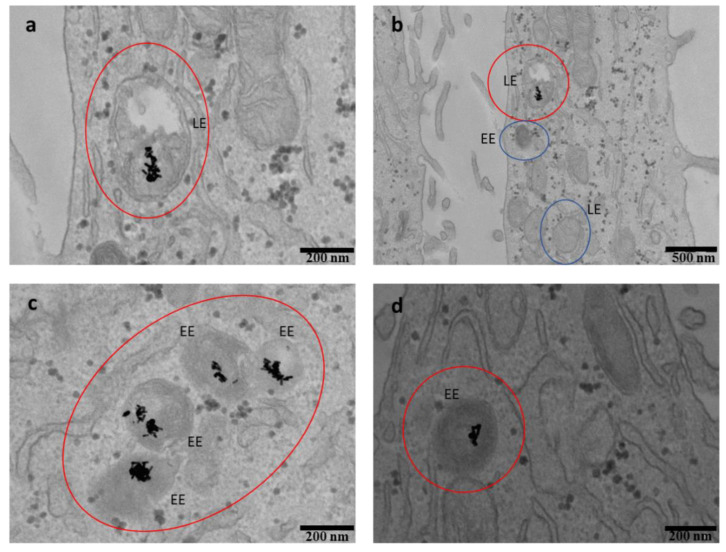
TEM images of HeLa cells after treatment with AuNR-BSA-r_8_ incubated for 24 h (**a**–**d**). Red circles show the internalized AuNPs. EE: early endosomes and LE: late endosomes. Blue circles showing examples of vacant LE or EE.

**Figure 9:**
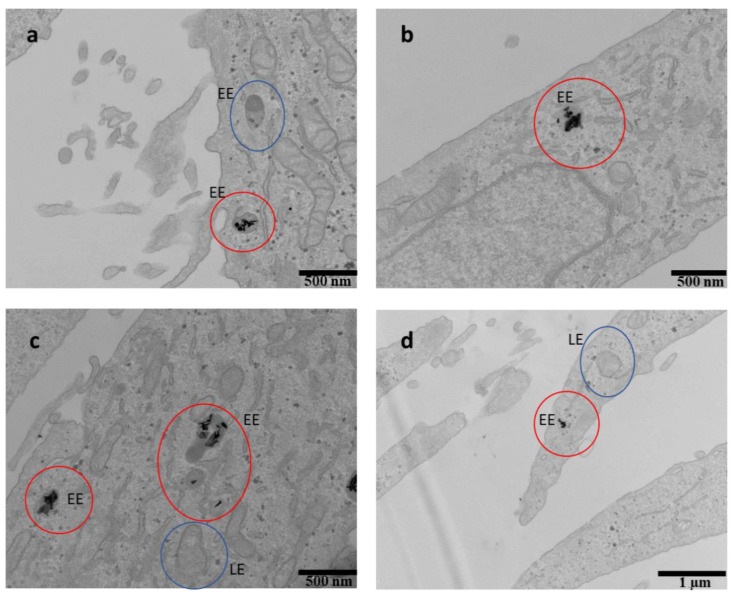
TEM images of HeLa cells after treatment with AuNPr-BSA-r_8_ incubated for 24 h (**a**–**d**). Red circles show the internalized AuNPs. EE: early endosomes and LE: late endosomes. Blue circles showing examples of vacant LE or EE.

The internalization of the BSA-A565-r_8_-coated AuNPs was assessed after 1 h and 24 h of incubation. Supplementary Section 6 (Figures S7 and S8) shows internalization at 1 h, indicating that the nanoconstructs started interacting with the cell membranes at this time, to become ready for the internalization process. At 24 h, in contrast, internalization of both AuNRs-BSA-A565-r_8_ and AuNPr-BSA-A565-r_8_ was considerable and observed under the studied conditions, as shown in Figure 8 and Figure 9.

The TEM images allowed determination of the precise location of the AuNPs in the cells. Both AuNRs and AuNPrs-based nanocarriers seemed to be located inside the cells and accumulated into vesicles in the cytoplasm, in agreement with the proposed endocytic uptake [29,51,59,88]. According to Liu et al. [89], the vesicles were labeled considering their size as early endosomes (200 nm diameter, EE) and late endosomes (500 nm diameter, LE). In Figure 8 and Figure 9, vacant EE and LE as well as the EE and LE where AuNPs are located were pointed out (blue and red circles, respectively). An average of 15 ± 5 AuNRs/vesicle and 18 ± 6 AuNPrs/vesicle were counted for AuNRs-BSA-A565-r_8_ and AuNPr-BSA-A565-r_8_, respectively (statistics of 30 vesicles from the TEM images). 

## 4. Conclusions

In this work, we tested the internalization abilities of two novel nanoconstructs based on anisotropic AuNPs with different surface and charge, AuNRs and AuNPrs coated with BSA in the presence and absence of a CPP (r_8_), improving AuNP internalization in the presence of r_8_. To the best of our knowledge, this is the first report that describes the coating of AuNRs and AuNPrs (with absorption in the first biological window) with BSA functionalized with r_8_ for improved cell internalization. These results point to the ability to improve the biocompatibility and internalization of nanoconstructs into cells by using endogenous proteins and CPPs. Nevertheless, as a further step, it would also be interesting to add targeting agents to the proposed nanoconstructs to increase their selectivity.

## Figures and Tables

**Table 1:** Physicochemical parameters of the synthesized nanoparticles and nanoconstructs.

Samples	Long. λ_max_ (nm)	Hydrodynamic Diameter (nm)		PDI	Z Potential (mV)
		Transversal	Longitudinal		
AuNR	775	2 ± 1	59 ± 3	0.5	45 ± 3
AuNR-BSA	757	3 ± 1	68 ± 3	0.5	−21 ± 1
AuNR-BSA-A565-r_8_	750	6 ± 1	79 ± 3	0.5	−17 ± 1
AuNPr	865	4 ± 1	68 ± 4	0.4	−31 ± 3
AuNPr-BSA	845	10 ± 2	142 ± 5	0.5	−14 ± 1
AuNPr-BSA-A565-r_8_	829	12 ± 2	142 ± 5	0.5	−18 ± 1

## Data Availability

Not applicable.

## Supplementary Materials

The following are available online at https://www.mdpi.com/article/10.3390/pharmaceutics13081204/s1: Figure S1: Structure of the Br-CH_2_-r_8_ peptide, Figure S2: UPLC trace (a) and MS spectra (b) of Br-r_8_ (BrCH2CO-r_8_), Figure S3: Microscopy characterization of AuNRs: a, b. representative TEM images, scale 200 nm, c. Aspect ratio frequency distri-bution (length/width), statistics of at least 50 AuNRs, Figure S4: Microscopy characterization of AuNPrs: a, b. representative TEM images, scale 200 nm, c. Edge length (nm) frequency distribution (length/width), statistics of at least 50 AuNPrs, Figure S5: Characterization of BSA-r_8_ capped AuNPs after 24 h, DLS and Zeta potential, Figure S6: Flow cytometry of Hela cells incubated by 24 h with: a. water, b. AuNR-BSA-r_8_ 1 nM in AuNRs, c. AuNPr-BSA-r_8_ 1 nM AuNPrs, Figure S7: TEM images of HeLa cells after treatment by AuNR-BSA-r_8_ incubated by 1 h, Figure S8: TEM images of HeLa cells after treatment by AuNPr-BSA-r_8_ incubated by 1 h, Table S1: Amino acid analysis of BSA-r8 after acid digestion, using ϒ-aminobutiric acid as standard (aaba).
