# Supplementary material for: Oligoarginine Peptide Conjugated to BSA Improves Cell Penetration of Gold Nanorods and Nanoprisms for Biomedical Applications
Source: Pharmaceutics. 2021 Aug 5;13(8):1204. doi: 10.3390/pharmaceutics13081204 (PMC8400532; doi:10.3390/pharmaceutics13081204)
Supplement: Supplementary file 1 [file pharmaceutics-13-01204-s001.zip › pharmaceutics-1305346-supplementary.pdf]

pharmaceutics

# Supplementary Materials: Oligoarginine Peptide Conjugated to BSA Improves Cell Penetration of Gold Nanorods and Nanoprisms for Biomedical Applications

Karen Bolaños, Macarena Sánchez-Navarro, Andreas Tapia-Arellano, Ernest Giralt, Marcelo J. Kogan and Eyleen Araya

## 1. BSA functionalization by r8 and AuNPs coating by BSA-r8

For this study, a D-octaarginine peptide (r8, Figure S1), with a Br terminal was synthesized and used to functionalize the BSA protein.

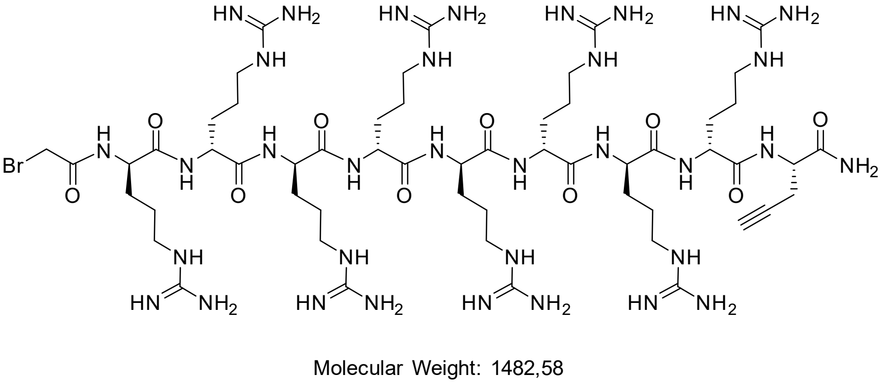


**Figure S1.** Structure of the Br-$CH_2$-r8 peptide.

The peptide was obtained with high purity (> 95%), and a characteristic mass spectrum, as shown in Figure S2.

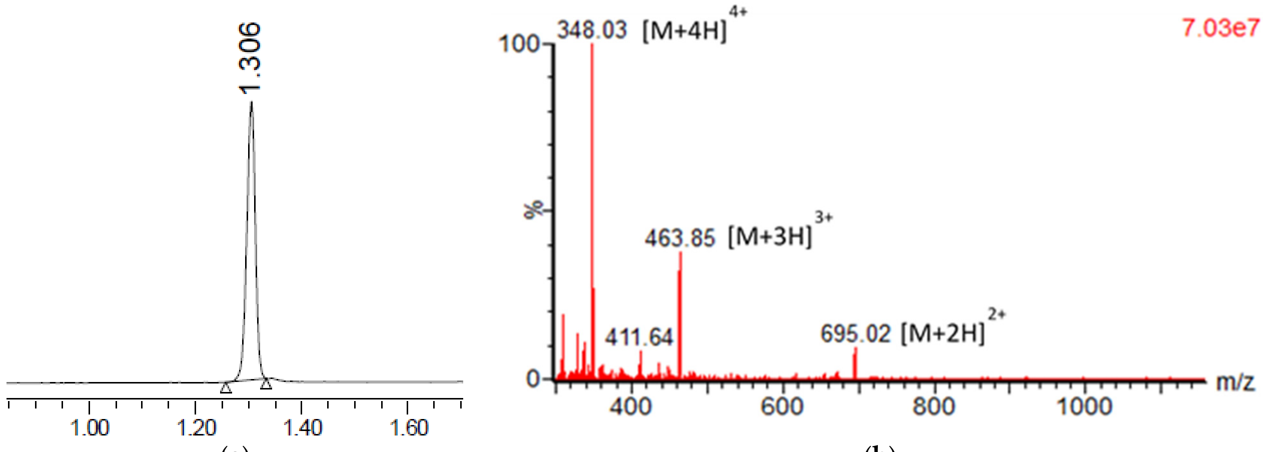


(a) (b)

**Figure S2.** UPLC trace (a) and MS spectra (b) of Br-r8 (Br$CH_2$CO-r8). UPLC chromatogram was recorded at 220 nm in a 2 min linear gradient from 0 to 30% of MeCN (0.036% TFA) in $H_2O$ (0.045% TFA). $M_{cal}$: 1387.48; $M_{found}$: 695.02 $[M+2H]^{2+}$; 463.85 $[M+3H]^{3+}$; 348.03 85 $[M+4H]^{4+}$.

## 2. BSA functionalization by A565 and r8

The r8 was synthesized with a Br terminal and BSA was functionalized following a 2-Iminothiolane strategy on the Br terminal of the peptide. Uniprot (P02769, Bos Taurus) for BSA sequence was consulted to know the theoretical number of Arg in a BSA mole-

cule, resulting in 26 Arg/BSA molecule. For amino acid analysis, 1.5 × $10^{-3}$ mM of BSA were added, equivalent to 3.9 × $10^{-2}$ mM of arginine. Therefore, the arginine excess was calculated subtracting the quantified amount of Arg (Table S1) from the theoretical amount of Arg in the sample (0.07 mM–0.039 mM = 0.031 mM). The arginine excess is translated to 3.64 × $10^{-3}$ mM of $r_8$, as follows:

$$r_8 amount = \frac{Arginine\ excess}{\frac{molar\ mass\ of\ arginine}{molar\ mass\ of\ r_8}} = \frac{0.031 mM}{\left(\frac{1482.6\ g/mol}{174.2\ g/mol}\right)} = 3.64 \times 10^{-3} mM\ r_8$$

The ratio $r_8$/BSA was calculated as 2.4 (3.64 × $10^{-3}$ mM $r_8$/1.5 × $10^{-3}$ mM BSA). The BSA-$r_8$ was subsequently labeled by an Atto-565 NHS-ester red fluorophore with a DOL of 0.66 mol Atto/mol BSA for the detection on fluorescence techniques, as previously described.

**Table S1.** Amino acid analysis of BSA-$r_8$ after acid digestion, using ϒ-aminobutiric acid as standard (aaba).

| Peak Name | RT (min) | Amount (mM) | Number of residues in protein |
|---|---|---|---|
| asp | 21.326 | 0.081 | 54 |
| ser | 23.120 | 0.053 | 31 |
| glu | 23.638 | 0.134 | 79 |
| gly | 25.041 | 0.031 | 17 |
| his | 25.328 | 0.039 | 17 |
| arg | **27.133** | **0.07** | **26** |
| thr | 27.500 | 0.072 | 34 |
| ala | 28.118 | 0.098 | 48 |
| pro | 29.263 | 0.06 | 28 |
| aaba | 30.504 | 0.103 | 0 |
| tyr | 32.417 | 0.023 | 21 |
| val | 33.292 | 0.067 | 38 |
| lys | 35.959 | 0.112 | 60 |
| ile | 37.030 | 0.029 | 15 |
| leu | 37.668 | 0.121 | 64 |
| phe | 39.453 | 0.052 | 30 |

## 3. TEM images of AuNRs and AuNPrs

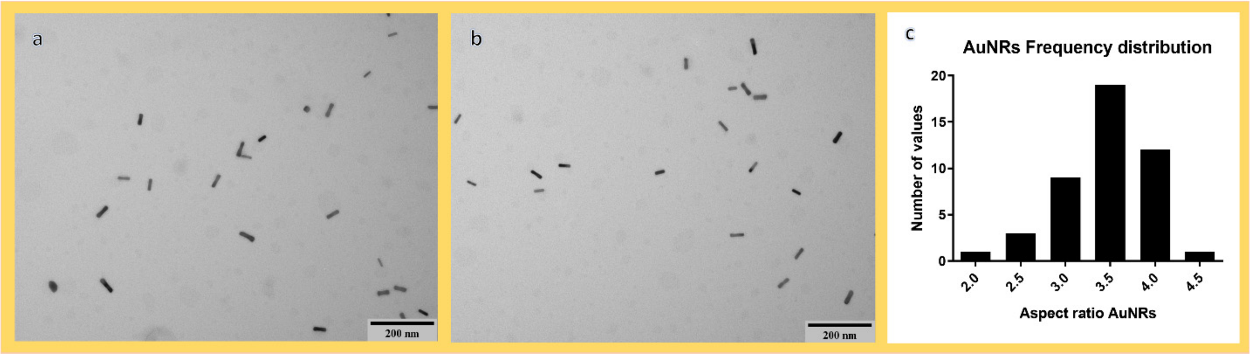


**Figure S3.** Microscopy characterization of AuNRs: **a, b.** representative TEM images, scale 200 nm, **c.** Aspect ratio frequency distribution (length/width), statistics of at least 50 AuNRs. Average population of 95% AuNRs.

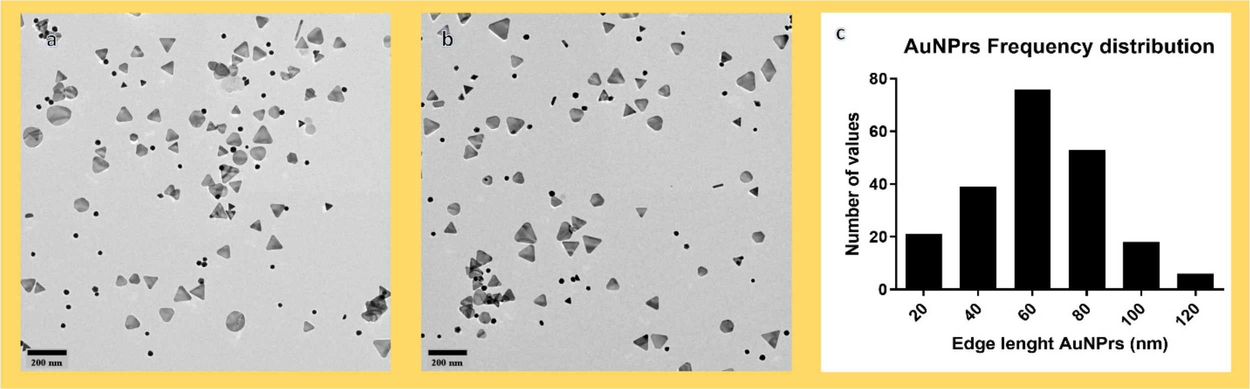


**Figure S4.** Microscopy characterization of AuNPrs: **a, b. r**epresentative TEM images, scale 200 nm, **c**. Edge length (nm) frequency distribution (length/width), statistics of at least 50 AuNPrs. Average population of 78% AuNPrs.

## 4. Stability of AuNR-BSA-$r_8$ and AuNPr-BSA-$r_8$ after 30 days

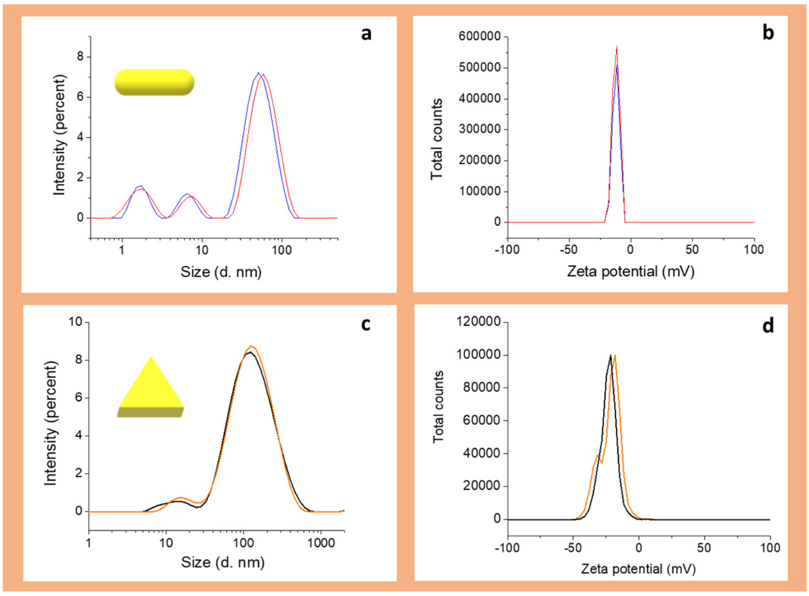


**Figure S5.** Characterization of BSA-$r_8$ capped AuNPs after 24 h, DLS and Zeta potential. **a, b**. AuNR-BSA- $r_8$ blue line t = 0, red line t = 30 days. **c,d**. AuNR-BSA- $r_8$ orange line t = 0, black line t = 30 days. Acquired in PBS pH = 7.

## 5. Flow cytometry of HeLa cells after treatment with AuNR-BSA-$r_8$ and AuNPr-BSA-$r_8$

HeLa cells (1 × $10^4$ cells/well) were seeded in pretreated 96 wells plates and allowed to attach at 37 °C for 24 h. Subsequently, the medium was removed, and a fresh medium was added. The treatments were added and allowed to incubate for 24 h, 1nM AuNR-BSA-$r_8$, 1 nM AuNPr-BSA-$r_8$. After this time, the cells were washed, trypsinized and collected in a plastic tube for flow cytometry analysis in a Gallios beckman coulter flow cytometer. HOESCHT was added to the samples (5 µL/mL) at least 10 min before the analysis. Dead cells are positive for the dye and identified in the upper region of the flow cytometry images.

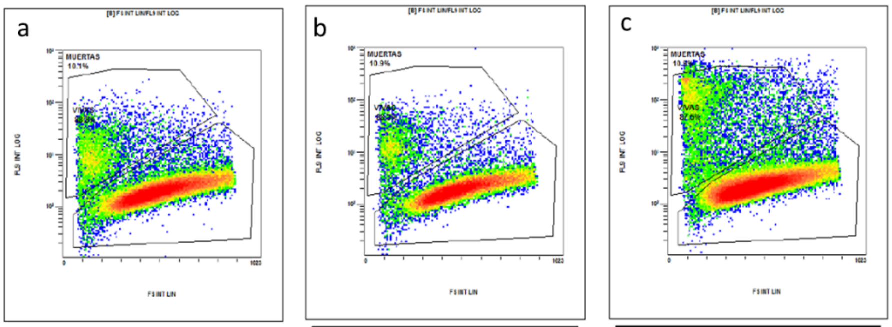


[B] FS INT LIN/FL9 INT LOG

| Region | Number | %Gated | X-Mean |
|---|---|---|---|
| ALL | 46457 | 100.00 | 461 |
| MUERTAS | 4680 | 10.07 | 204 |
| VIVAS | 41318 | 88.94 | 492 |

[B] FS INT LIN/FL9 INT LOG

| Region | Number | %Gated | X-Mean |
|---|---|---|---|
| ALL | 27631 | 100.00 | 454 |
| MUERTAS | 3002 | 10.86 | 198 |
| VIVAS | 24389 | 88.27 | 487 |

[B] FS INT LIN/FL9 INT LOG

| Region | Number | %Gated | X-Mean |
|---|---|---|---|
| ALL | 77258 | 100.00 | 435 |
| MUERTAS | 8401 | 10.87 | 241 |
| VIVAS | 67636 | 87.55 | 460 |

**Figure S6.** Flow cytometry of Hela cells incubated by 24 h with: a. water, b. AuNR-BSA-r8 1 nM in AuNRs, c. AuNPr-BSA-r8 1 nM AuNPrs. Apoptotic cells are identified by positive fluorescence in the upper region by DAPI labeling.

For this study, death cells were identified by positive fluorescence in the upper region due to DAPI labeling. As shown in figure S7, no significative effect was observed at 24 h of treatment with 1nM AuNR-BSA-r8, 1 nM AuNPr-BSA-r8 in the studied conditions, by comparing the treated cells with the control.

## 6. Internalization of AuNR-BSA-A565-r8 on HeLa cells at 1h

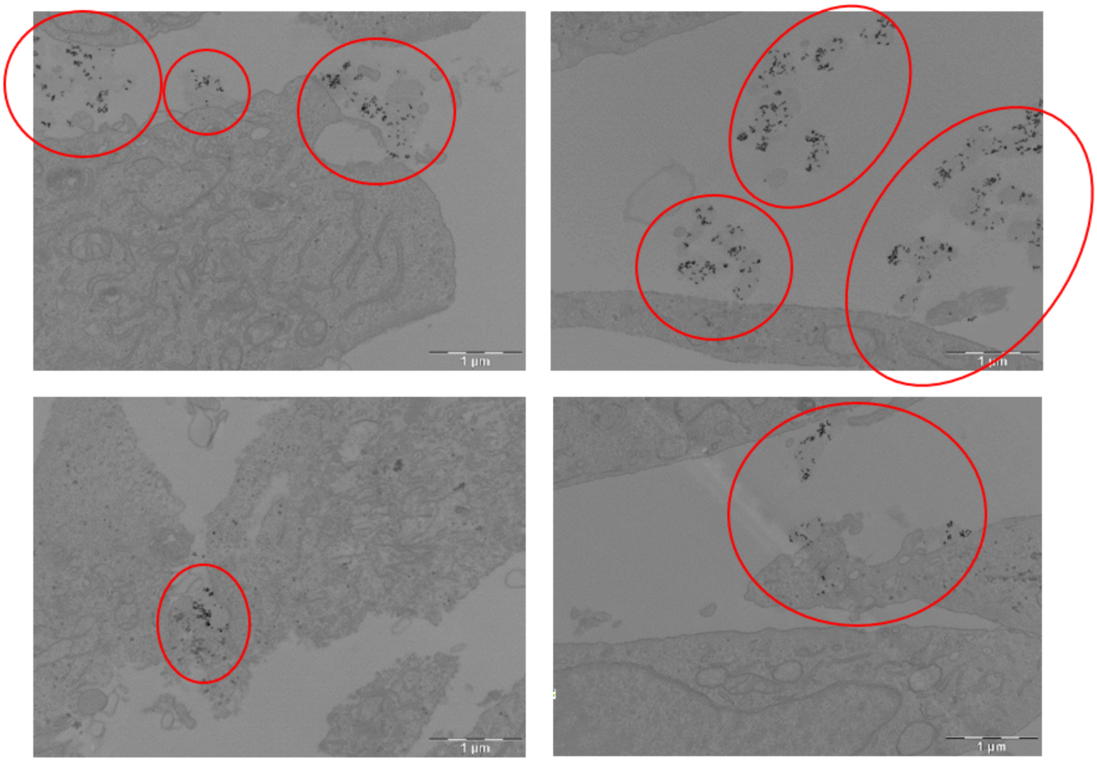


**Figure S7.** TEM images of HeLa cells after treatment by AuNR-BSA-r8 incubated by 1h. Scale 1 µm. Red circles showing the AuNRs.

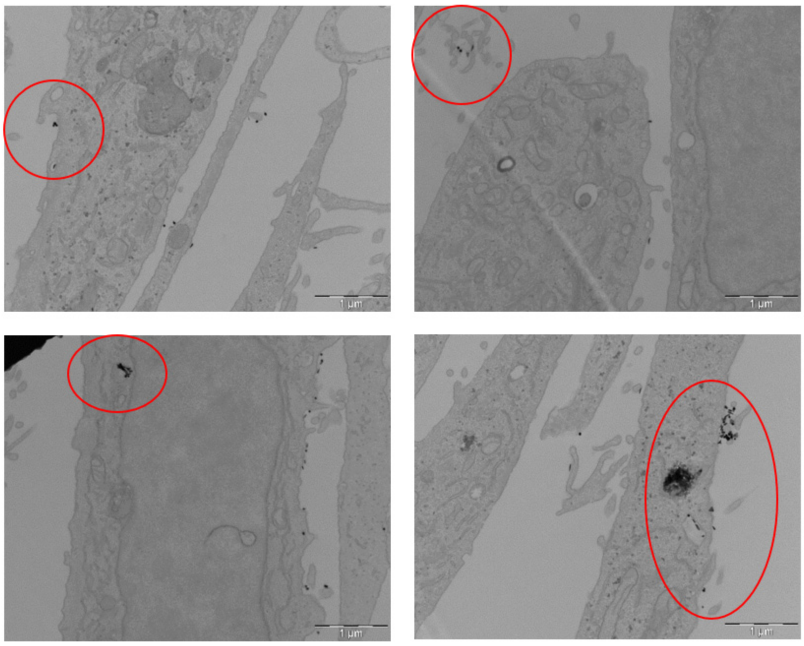

**Figure S8.** TEM images of HeLa cells after treatment by AuNPr-BSA-$r_8$ incubated by 1h. Scale 1 µm. Red circles showing the AuNPrs.
